# An unexpected primary squamous cell carcinoma of the left colon: a rare case report

**DOI:** 10.1093/jscr/rjae680

**Published:** 2024-11-26

**Authors:** Charlotte Cornwell, Tae Jun Kim, Chris M Byrne

**Affiliations:** Department of Colorectal Surgery, Royal Prince Alfred Hospital, 50 Missenden Road Camperdown, NSW 2050, Australia; Department of Colorectal Surgery, Royal Prince Alfred Hospital, 50 Missenden Road Camperdown, NSW 2050, Australia; Surgical Outcomes and Research Centre (SOuRCe), Sydney Local Health District, Royal Prince Alfred Hospital, Level 9, Building, 89 Missenden Rd, Camperdown, NSW 2050, Australia; Department of Colorectal Surgery, Royal Prince Alfred Hospital, 50 Missenden Road Camperdown, NSW 2050, Australia; Surgical Outcomes and Research Centre (SOuRCe), Sydney Local Health District, Royal Prince Alfred Hospital, Level 9, Building, 89 Missenden Rd, Camperdown, NSW 2050, Australia

**Keywords:** squamous cell carcinoma, colorectal cancer, case report

## Abstract

Colorectal cancer is a common cancer with a large burden of disease. Adenocarcinomas account for majority of colorectal cancers, arising from glandular epithelium. Other malignancies including neuroendocrine, adenosquamous, spindle cell and squamous cell carcinomas (SCCs) are seldomly encountered. Primary colorectal SCC was first reported in 1919 and is particularly rare. It is difficult to manage as patients present late, with locally invasive or metastatic disease. We present the case of a woman in her 40s with a previously resected sigmoid adenocarcinoma and a new splenic flexure mass. Histopathology revealed an SCC without evidence of extra-colonic disease. The patient underwent resection with clear margins, however, did not tolerate systemic adjuvant treatment and developed local recurrence within twelve months. We add our patient’s case to the small compilation of cases of primary colorectal SCC along with a summary of its clinical and histological characteristics, strategies in management and considerations for future research.

## Introduction

Gastrointestinal SCCs occur most commonly in the oesophagus or anus. Primary colorectal SCC however is rare and carries a poor prognosis. Patients present with advanced disease stage and respond poorly to systemic therapies with high rates of recurrence. No treatment guidelines exist due to its rarity [1]. There are several proposed pathological mechanisms including transformation of pluripotent stem cells or repeated mucosal injury, however the development of squamous epithelial tumours in tissue which typically gives rise to glandular epithelial neoplasms remains perplexing [2]. In this article we report a case of locally advanced primary colonic splenic flexure SCC following a previous anterior resection for adenocarcinoma.

## Case report

**Figure 1:**
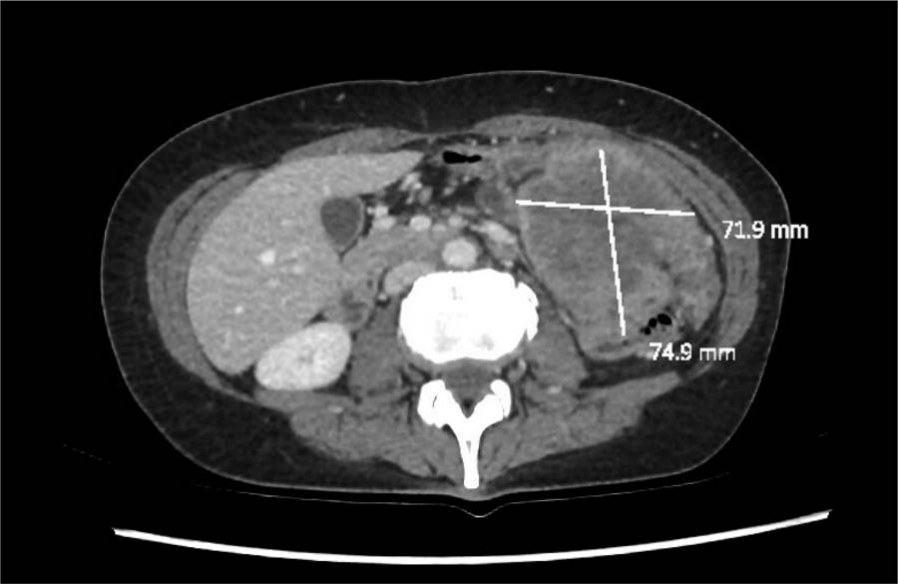
Axial slice of a CT demonstrating a large heterogenous mass in the left upper quadrant.

**Figure 2:**
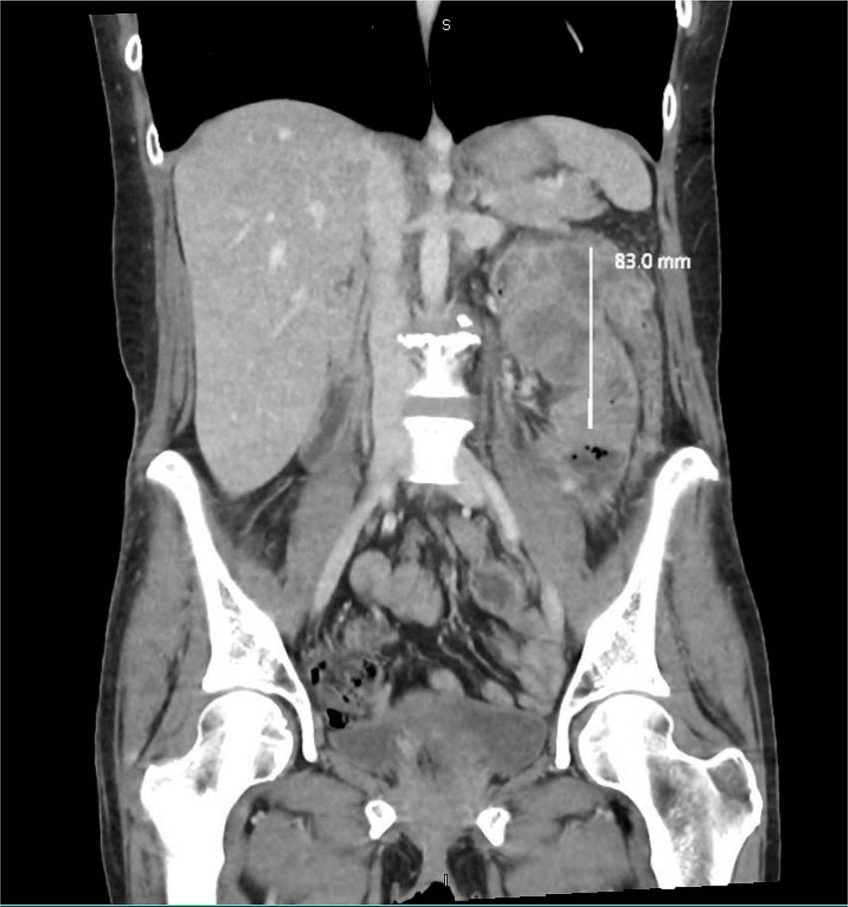
Coronal slice of a CT demonstrating necrotic mass arising from the large bowel with significant wall thickening.

We report a 47-year-old Indigenous woman presenting to a rural centre with nausea, vomiting and abdominal pain. She denied haematochezia or weight loss. Medical history was significant for a previous anterior resection for a T4bN2bM1 (19/44 nodes positive) sigmoid adenocarcinoma ten years prior. The patient received adjuvant chemotherapy with capecitabine at that time, however had poor compliance, did not attend follow up appointments or receive surveillance imaging or colonoscopies. There was no family history of malignancy. There was significant alcohol, tobacco and marijuana use. On examination, she had upper abdominal tenderness without a palpable mass. CEA was 4.6 ug/L, and there was normocytic anaemia. Computed tomography revealed a 7 × 9 × 7cm non-obstructing necrotic splenic flexure mass with adjacent small bowel invasion, however no recurrence at the site of previous anterior resection (Figs 1 and 2). Colonoscopy showed an ulcerated circumferential mass which was unable to be traversed. The patient was transferred to our metropolitan tertiary centre for ongoing management.

The patient was optimised preoperatively with parenteral nutrition, electrolyte replacement and blood transfusions. Positron emission tomography demonstrated no extra-abdominal sites of disease to suggest an alternative primary or distant metastasis. Laparotomy revealed invasion into the adjacent jejunum and retroperitoneum with suspicious mesenteric lymph nodes, however the stomach and pancreas were uninvolved. There were no liver or peritoneal metastases and the previous anastomosis appeared healthy. A subtotal colectomy and enbloc jejunectomy with primary duodenojejunostomy and formation of an abcarian ileocolostomy was performed. The patient recovered well and was transferred to the referring hospital on postoperative day thirteen for convalescent care. Histopathology showed a poorly differentiated SCC with no evidence of adenocarcinoma and a malignant entero-colic fistula to the jejunectomy specimen. Three of thirty-two lymph nodes were positive and resection margins were clear. The lesion was staged as pT4bN1b. After multidisciplinary discussion, she received adjuvant chemotherapy with carboplatin and paclitaxel. However, due to severe toxicity further cycles were abandoned.

Eight months later, surveillance scanning revealed a new retroperitoneal mass in the surgical bed. A repeat PET scan demonstrated no distant metastases. The case was discussed at a multidisciplinary meeting and the recurrence deemed resectable. However, whilst awaiting the procedure the patient developed melaena and anaemia. Endoscopy revealed a large duodenal ulceration, and she was urgently transferred to our centre for expedited surgery. Serial imaging only three weeks later demonstrated rapid growth of the recurrence with encasement of the superior mesenteric vessels and pancreatic invasion. This was deemed unresectable and she was transferred to the referring hospital for palliative radiotherapy.

## Discussion

Primary colorectal SCC is rare with an incidence of 0.25–0.1 per 1000 colorectal cancers and only 150 cases reported in literature [1]. Its aetiology, pathophysiology, presentation, management, and prognosis remain poorly defined. The disease is usually seen in males in the fifth decade of life in the rectosigmoid or on the right side. Left-sided cancers are even more infrequent with only three other cases ever reported in the literature [3, 4].

Gastrointestinal SCCs arise from squamous epithelium and are therefore typically found in the oesophagus or anus. The glandular epithelium of the colon typically gives rise to adenocarcinomas. Multiple theories have been proposed to explain this. One theory suggests metaplasia of glandular epithelial cells into squamous epithelium secondary to mucosal insults like radiation, infection, inflammatory bowel disease, or carcinogens. Alternatively, there are endodermal pluripotent stem cells in the colon that can differentiate and undergo malignant transformation, also explaining the occurrence of small cell, carcinoid and neuroendocrine tumours of the colon. There may be an association with Schistosomiasis and human papilloma virus infection although this remains to be proven [5].

Colorectal SCCs present similarly to adenocarcinomas with pain, weight loss, haematochezia and altered bowel habits, or emergently with perforation, bleeding or obstruction. There is a reported diagnostic delay of six to twelve months, with synchronous liver, lung or bone metastases in approximately 50% of cases [6, 7].

Diagnosis of primary colorectal SCC can only be made if several conditions are true. Firstly, metastasis to or direct invasion of the colon or rectum must be excluded. Although rare, there are reports of colorectal metastases from SCCs of the tongue, penis and skin [8, 9]. Secondly, there must be histopathological features consistent with SCC with no glandular component. Thirdly, there must be no fistula tract from another squamous-lined epithelial surface to the bowel. Lastly, the SCC cannot extend from the anal squamous epithelium into the colon or rectum [10]. SCC Antigen is a tumour marker used in lung, gynaecological, oesophageal and head and neck SCCs, although it is not used in primary colorectal SCC and was not measured in our patient [5].

Consensus guidelines do not exist for primary colorectal SCC. Principles in management stem from those employed for adenocarcinoma, and resection forms the cornerstone of management. Adjuvant chemoradiation regimens similar to those used for SCCs arising from other sites have been trialled and are rarely effective, with fewer than five reports of patients who responded to therapy [1]. Immunotherapy was trialled in combination with chemotherapy in one case with minimal response [11].

Prognosis for colorectal SCC is worse than adenocarcinoma, owing to advanced stage at diagnosis and poor response to systemic therapy. Most cases of SCC appearing in the literature report recurrence or death within twelve months [4, 12].

## Conclusion

Primary colorectal SCC is an uncommon tumour with uncertain pathogenesis. The mainstay of management is surgical resection, with no clear role for systemic therapy. The disease is so seldom encountered that minimal evidence exists to inform practice, and most patients experience poor outcomes despite these measures. Most reported cases describe aggressive disease which rapidly recurs. This indicates the need for radical resection, stringent follow up and additional research to inform systemic therapies. Colorectal SCC often comes as a histopathological surprise, and when encountered should be approached with caution given its aggressiveness and poorly defined management principles.
